# Exploring the Deoxy-D-xylulose-5-phosphate Synthase Gene Family in Tomato (*Solanum lycopersicum*)

**DOI:** 10.3390/plants12223886

**Published:** 2023-11-17

**Authors:** Xueni Di, Manuel Rodriguez-Concepcion

**Affiliations:** Institute for Plant Molecular and Cell Biology (IBMCP), CSIC—Universitat Politècnica de València, 46022 Valencia, Spain

**Keywords:** carotenoid, DXS, heterodimer, isoprenoid, MEP pathway, tomato

## Abstract

Isoprenoids are a wide family of metabolites including high-value chemicals, flavors, pigments, and drugs. Isoprenoids are particularly abundant and diverse in plants. The methyl-D-erythritol 4-phosphate (MEP) pathway produces the universal isoprenoid precursors isopentenyl diphosphate and dimethylallyl diphosphate in plant plastids for the downstream production of monoterpenes, diterpenes, and photosynthesis-related isoprenoids such as carotenoids, chlorophylls, tocopherols, phylloquinone, and plastoquinone. The enzyme deoxy-D-xylulose 5-phosphate synthase (DXS) is the first and main rate-determining enzyme of the MEP pathway. In tomato (*Solanum lycopersicum*), a plant with an active isoprenoid metabolism in several tissues, three genes encode DXS-like proteins (SlDXS1 to 3). Here, we show that the expression patterns of the three genes suggest distinct physiological roles without excluding that they might function together in some tissues. We also confirm that SlDXS1 and 2 are true DXS enzymes, whereas SlDXS3 lacks DXS activity. We further show that SlDXS1 and 2 co-localize in plastidial speckles and that they can be immunoprecipitated together, suggesting that they might form heterodimers in vivo in at least some tissues. These results provide novel insights for the biotechnological use of DXS isoforms in metabolic engineering strategies to up-regulate the MEP pathway flux.

## 1. Introduction

Isoprenoids are one of the largest and most structurally diverse family of natural products, encompassing tens of thousands of compounds. Most of these compounds are produced by plants and many are economically relevant as pigments, nutrients, flavors, chemicals, or drugs for commercial applications [1,2]. All isoprenoids are derived from isopentenyl diphosphate (IPP) and its double-bond isomer dimethylallyl diphosphate (DMAPP). These universal isoprenoid precursors are synthesized by the mevalonate (MVA) pathway in eukaryotes and most archaea and by the methyl-D-erythritol 4-phosphate (MEP) pathway in most bacteria. Unlike most organisms, plants use both pathways: the MVA pathway produces cytosolic isoprenoid precursors to synthesize sterols, sesquiterpenes, and polyterpenes, whereas the MEP pathway functions in plastids and produces IPP and DMAPP for monoterpenes, diterpenes, and photosynthesis-related isoprenoid compounds such as carotenoids, chlorophylls, tocopherols, phylloquinone, and plastoquinone [2,3]. The first enzyme of the MEP pathway is deoxy-D-xylulose 5-phosphate (DXP) synthase (DXS), which catalyzes the biosynthesis of DXP from pyruvate and glyceraldehyde 3-phosphate using thiamine diphosphate (TPP) as a cofactor. Metabolic control analyses have shown that DXS is the MEP pathway enzyme with the highest flux control coefficient in bacteria or plant systems [4,5], consistent with the conclusion that it catalyzes the main rate-determining step of the pathway. In agreement with this central role in the regulation of the MEP pathway flux, the production of MEP-derived isoprenoid end-products can be enhanced just by increasing DXS levels [6,7,8]. Also consistently, the expression of DXS-encoding genes is activated when an extra supply of IPP and DMAPP is needed for carotenoid biosynthesis during seedling de-etiolation in *Arabidopsis thaliana* [9] or fruit ripening in pepper (*Capsicum annuum*) [10] and tomato (*Solanum lycopersicum*) [6].

DXS is a highly conserved protein in bacteria and plants. The crystallization of DXS proteins from *Escherichia coli* [11] and *A. thaliana* [12] led to the conclusion that the active enzyme is a dimer. DXS protein monomers contain three domains (I, II, and III), similar to related TPP-dependent enzymes such as transketolase and the E1 subunit of pyruvate dehydrogenase. In the enzymatically active DXS dimer, domain I of one monomer is directly located above domains II and III of the same monomers, and two monomers with the same structure are arranged side by side. The active site is located at the interface of domains I and II in the same monomer. In the dimer, the TPP cofactor is buried in the active site present in each monomer with the C-2 atom of its thiazolium ring exposed to a pocket that is the substrate binding site [11].

While bacteria typically contain a single DXS-encoding gene, plants usually have small gene families encoding different DXS proteins, which can be grouped in three distinct classes based on sequence homology: class 1, 2, and 3 [13,14,15,16,17]. While class 1 DXS enzymes mostly play housekeeping roles, those belonging to class 2 are typically involved in the synthesis of specialized (or secondary) isoprenoids for defense responses and signaling. Recent phylogenetic studies suggest that class 3 DXS isoforms emerged later than class 1 and class 2 to play unknown functions unrelated to isoprenoid biosynthesis [17]. The tomato genome contains three DXS-encoding genes, one of each class: *SlDXS1* (Solyc01g067890), *SlDXS2* (Solyc11g010850), and *SlDXS3* (Solyc08g066950) [13,17,18,19,20]. Mutants defective in SlDXS1 exhibit albinism and are unable to develop, indicating a non-redundant role of SlDXS2 and SlDXS3 and supporting a major role for SlDXS1 in the production of essential (e.g., photosynthesis-related) isoprenoids [19]. SlDXS1 also supplies the precursors for the production of carotenoids that takes place during fruit ripening [20]. Mutants defective in SlDXS2 are not available yet, but the partial silencing of *SlDXS2* expression in tomato by RNAi resulted in decreased levels of the monoterpene β-phellandrene and increased the levels of two sesquiterpenes in trichomes [18]. Because monoterpenes are derived from the plastidial MEP pathway but sesquiterpenes are usually derived from the cytosolic MVA pathway, it was proposed that SlDXS2 might somehow alter ratios of MEP to MVA pathway allocation [18]. No experimental data are available for SlDXS3.

Here, we simultaneously studied the three tomato DXS isoforms (SlDXS1, SlDXS2, and SlDXS3) at gene expression, protein localization and structure, and enzyme activity levels. The results provide a new common framework that facilitates the functional comparison of the different isoforms, with the eventual goal of optimizing DXS activity for metabolic engineering approaches aimed at improving the content of plastidial isoprenoid products in plant (and bacterial) biofactories.

## 2. Results

### 2.1. DXS-Encoding Tomato Transcripts Are Differentially Expressed but Overlap in Some Tissues

**Figure 1:**
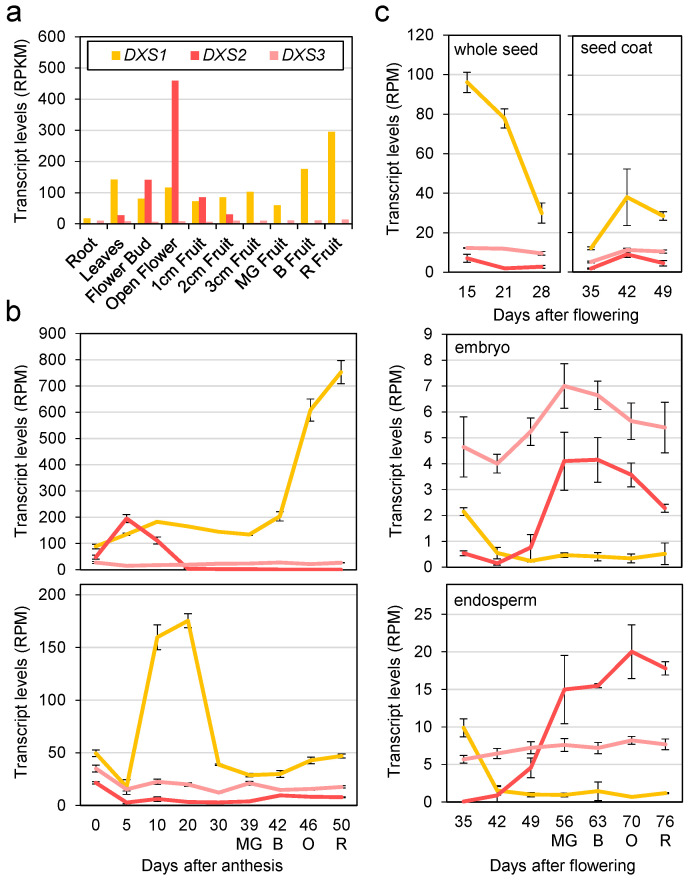
Expression profiles of *SlDXS1*, *SlDXS2* and *SlDXS3* genes in different tomato organs and tissues. (**a**) Transcript levels in samples taken from the tomato cultivar M82. Levels are represented as RPKM (reads per kb of transcript per million mapped reads). (**b**) Transcript levels in pericarp and seed tissues from M82 fruit expressed as RPM (reads per million mapped reads). (**c**) Transcript levels in seed tissues of the tomato cultivar MoneyMaker. Mean and standard deviation values are represented from RNAseq data retrieved from the Tomato eFP Browser (**a**,**c**) or the Tomato Expression Atlas (**b**). Colors in the line plots (**b**,**c**) represent the different DXS-encoding genes as indicated in the bar plot (**a**). Letters indicate approximate, color-based ripening stage to facilitate comparison among experiments: MG, mature green; B, breaker; O, orange; R, red.

Information on the expression patterns of the genes encoding SlDXS1, SlDXS2 and SlDXS3 is scattered in the literature. To provide a systematic view of these gene expression profiles, we collected publicly available information from the Tomato eFP browser (https://bar.utoronto.ca/efp_tomato/cgi-bin/efpWeb.cgi (accessed on 27 September 2023)) and the Tomato Expression Atlas (https://tea.solgenomics.net (accessed on 27 September 2023)) (Figure 1). In agreement with its housekeeping role, *SlDXS1* is expressed in all tomato organs and tissues, including roots, leaves, flowers, fruits, and seeds. In general, *SlDXS2* and *SlDXS3* are expressed at very low or undetectable levels, but there are some exceptions. *SlDXS2* is expressed at detectable levels in leaves and its expression in flowers is higher than that of *SlDXS1* (Figure 1a). This is in agreement with previous reports showing that *SlDXS2* is expressed at highest levels in leaf trichomes and flower organs (petals, sepals, stamens and pistils) [18]. Most interestingly, *SlDXS1* and *SlDXS2* are similarly co-expressed in young fruits (Figure 1a). Separate analysis of pericarp and seed samples showed that this overlapping pattern is mostly restricted to the pericarp (Figure 1b). Later on, during fruit growth to acquire their final size at the mature green (MG) stage and during the following stages of ripening, *SlDXS1* expression becomes predominant in the pericarp and the seeds (Figure 1b). In the pericarp, *SlDXS1* expression is strongly increased during ripening (Figure 1b), likely to supply the MEP-derived precursors needed for the synthesis of carotenoid pigments [6,20]. *SlDXS1* transcripts are much less abundant in seeds, where their levels peak at the stage when young fruits are actively growing (15–30 days after flowering or 10–20 days after anthesis) (Figure 1b,c). *SlDXS2* transcript levels in seed tissues are also low, but they are higher than those of *SlDXS1* in the embryo and in the endosperm, particularly after the mature seeds present in MG fruit begin to dry and acquire their dormancy during ripening (Figure 1c). Different from other isoforms, *SlDXS3* transcript levels are constitutively low in all organs and tissues. These results together support the conclusion that DXS isoforms may have mutually exclusive, non-redundant roles in most tissues but suggest a coordinated role for SlDXS1 and SlDXS2 in the pericarp of young fruit based on their overlapping expression pattern.

### 2.2. SlDXS1 and SlDXS2, but Not SlDXS3, Are True DXS Enzymes

**Figure 2:**
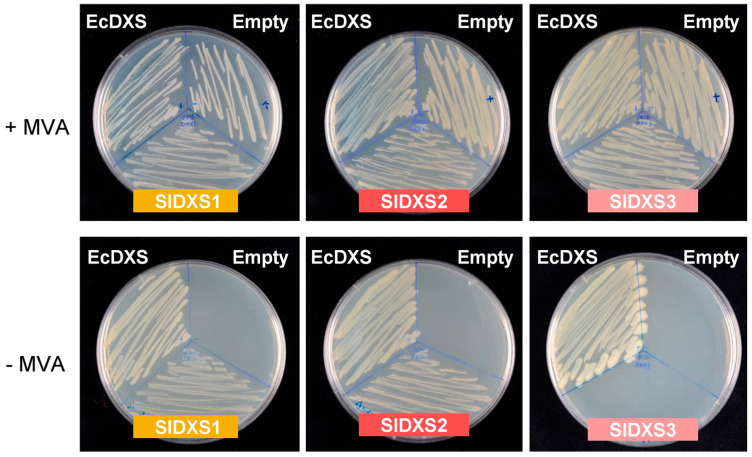
Complementation of DXS-deficient *E. coli* cells with tomato DXS sequences. DXS-deficient EcAB4-2 cells carrying a synthetic MVA operon were transformed with constructs encoding the indicated DXS sequences or an empty plasmid as a negative control. Transformants were then grown on LB plates either supplemented (+) or not (−) with MVA.

Plant DXS sequences belonging to class 1 and 2 have been shown to rescue the growth of DXS-deficient *E. coli* strains [14,15,18,20], whereas the DXS3 sequences from several species were unable to provide the DXS activity required in these complementation experiments [17]. To test the activity of the three tomato DXS isoforms in *E. coli* complementation experiments, we initially analyzed SlDXS1, SlDXS2 and SlDXS3 protein sequences with Target P [21] to estimate the length of their putative plastid-targeting motifs. Sequences lacking the predicted N-terminal plastid-targeting sequences were amplified from a tomato ripe fruit cDNA library using gene-specific oligonucleotides. Then, the amplified cDNAs were cloned in a pBluescript vector and the generated constructs were used to complement the lethal phenotype of the DXS-lacking *E. coli* strain EcAB4-2 [22]. The genome of this strain harbors a synthetic MVA operon that allows producing IPP and DMAPP when MVA is supplied to the growth medium, hence bypassing the lethal absence of DXS activity. EcAB4-2 transformants carrying the constructs with the tomato DXS sequences were grown in LB media either with or without MVA. As controls, we used an empty plasmid and a construct encoding the *E. coli* DXS enzyme (EcDXS). All transformed strains grew when MVA was supplied to the medium (Figure 2). The strain transformed with the EcDXS construct also grew in the absence of MVA (Figure 2). The same was observed when using the SlDXS1 and SlDXS2 constructs, whereas the strains harboring the SlDXS3 construct or an empty plasmid were unable to grow when MVA was not supplied. These results demonstrate that SlDXS1 and SlDXS2 are functional DXS enzymes, but SlDXS3 lacks DXS activity, at least when expressed in *E. coli*. 

**Figure 3:**
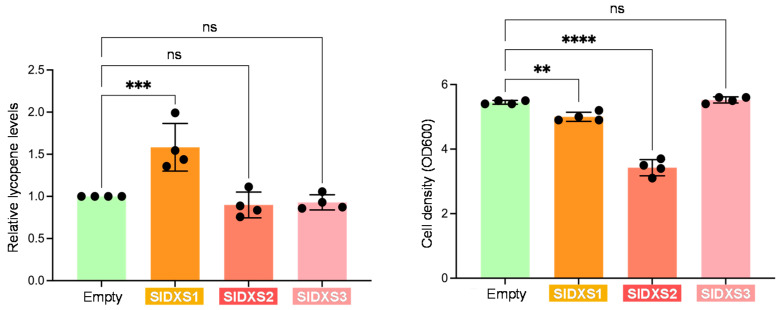
Lycopene production in *E. coli* cells harboring tomato DXS sequences. Lycopene content is represented normalized to cell density and relative to that in control cells transformed with an empty vector. Dots represent individual data points. Mean and standard deviation are also represented. Asterisks indicate statistically significant difference (one-way ANOVA with Dunnett’s multiple comparisons test; ****: *p*-value  ≤  0.0001; ***: *p*-value  ≤ 0.001; **: *p*-value  ≤ 0.01; ns: not significant).

As an alternative way to test the activity of the tomato DXS isoforms, we evaluated their capacity to activate the production of the red carotenoid lycopene in *E. coli* cells carrying the p421-LYC plasmid [23]. This construct contains a carotenoid operon from *Pantoea ananatis* that allows the production of lycopene from IPP and DMAPP. Therefore, enhanced production of IPP and DMAPP by an activated MEP pathway flux should result in higher lycopene levels. *E. coli* cells of the BL21(DE) strain were co-transformed with p421-LYC and the tomato DXS constructs used in the complementation experiments. Transformants were grown at 30 °C for 24 h and, after measuring optical density at 600 nm (OD600), cells were collected to quantify their lycopene content via acetone extraction followed by a measurement of absorbance at 472 nm. Lycopene levels were calculated by normalizing absorbance to cell density. Notably, the only DXS isoform able to promote lycopene production was SlDXS1 (Figure 3). SlDXS2 did not activate lycopene synthesis in *E. coli* cells, but it led to a decrease in cell density (Figure 3), suggesting that it might interfere with cell growth, e.g., by creating toxic aggregates. This result also suggests a differential folding or aggregation propensity of SlDXS1 and SlDXS2. SlDXS3 had no effect in lycopene accumulation or cell growth (Figure 3), further supporting the conclusion that this isoform is not a true DXS.

### 2.3. SlDXS3 Protein Lacks Structural Features Required for DXS Activity

**Figure 4:**
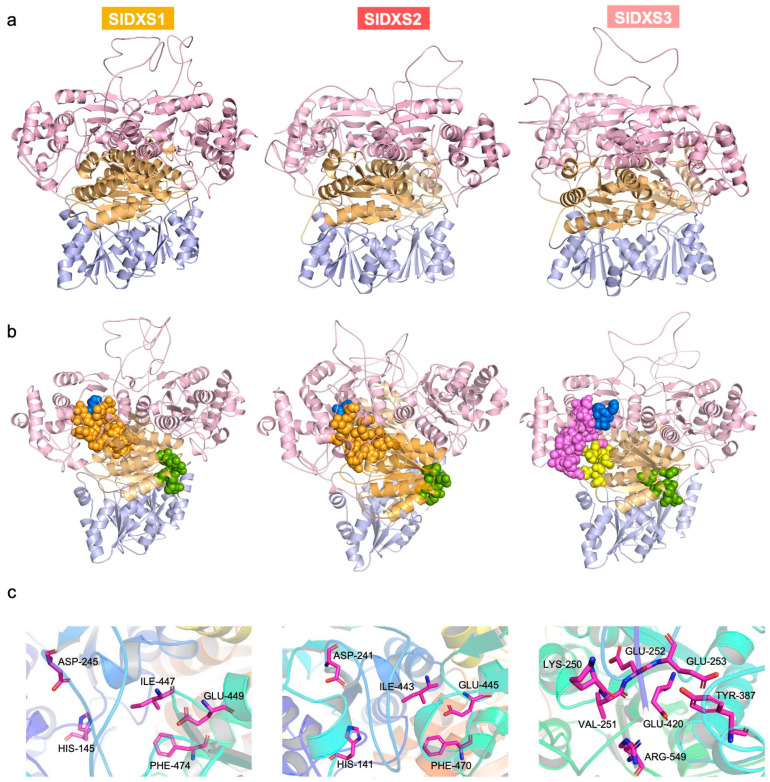
Modeling of tomato DXS protein structures. (**a**) SlDXS1, SlDXS2 and SlDXS3 protein structures modeled by Swiss-Model. The three domains in the monomers are distinguished by colors: pink for domain I, orange for domain II and purple for domain III. (**b**) Ligand binding sites predicted in SlDXS1, SlDXS2 and SlDXS3. The position of the sites is marked with colors: green and blue for the first and second sites in all three sequences, respectively (putatively involved in ion binding); orange for the third site in SlDXS1 and SlDXS2 (responsible for TPP and pyruvate binding); and purple and yellow for the third and fourth sites in SlDXS3 (predicted to bind FAD and pyruvate binding). (**c**) Conserved residues at the TPP binding site of SlDXS1 and SlDXS2 and residues at the pyruvate binding site of SlDXS3.

DXS crystal structures are difficult to obtain due to conformational changes during the catalytic cycle [24,25] and proteolytic degradation [11,26,27]. As an alternative to investigating structural differences among the three tomato DXS isoforms, we decided to exploit the available structural information for comparative protein structure modeling [28,29]. We used the cryo-electron microscopy structure of the *A. thaliana* DXS protein [12] as a template (7bzx.1.A) to generate the three-dimensional structures of SlDXS1, SlDXS2 and SlDXS3 with Swiss-Model [30] based on the target–template alignment using ProMod3 (Figure 4). Similar coverage to the target sequence was observed for the three tomato DXS proteins: 0.92 (72–706) for SlDXS1, 0.91 (68–702) for SlDXS2 and 0.92 (69–696) for SlDXS3 (Table 1). The sequence identity of SlDXS1, SlDXS2 and SlDXS3 with the template is 88.75%, 73.93%, and 58.24%, respectively, illustrating their sequence diversity. However, the overall structure was similar for the three isoforms (Figure 4a). The Global Model Quality Estimate (GMQE), which evaluates the combined properties from the target–template alignment and the template structure, was 0.72, 0.70 and 0.65 for SlDXS1, SlDXS2 and SlDXS3, respectively (Table 1), showing a good reliability of the prediction.

DXS enzymes use pyruvate and glyceraldehyde 3-phosphate as substrates to produce DXP with a loss of CO_2_ in a reaction that requires a divalent cation (typically Mg^2+^ or Mn^2+^) and uses TPP as a cofactor at the active site [11,31,32,33]. The physical interaction of DXS enzymes with their ligands (such as substrates or cofactors) is necessary for their catalytic activity. In order to identify ligand binding sites in SlDXS1, SlDXS2 and SlDXS3, the three protein models were uploaded to the 3D Ligand Site web server [34], which is one of the top-performing predictors of ligand binding sites according to the Critical Assessment of techniques for protein Structure Prediction (CASP8) [35]. Predicted binding sites for the TPP cofactor, the pyruvate substrate and ion ligands were found in SlDXS1 and SlDXS2 (Figure 4b). In the case of SlDXS3, binding sites were found for flavin- adenine dinucleotide (FAD), pyruvate and ion ligands, but not for TPP (Figure 4b). Na^+^ and K^+^ are predicted ion ligands at the first binding site of all three DXS proteins, whereas binding of Mg^2+^, Ca^2+^ and Mn^2+^ is predicted at the second binding site of SlDXS1. Only Mg^2+^ and Ca^2+^ binding were predicted for SlDXS2 and SlDXS3. 

Residues participating in TPP binding (His, Asp, Ile, Glu, and Phe) are highly conserved in DXS enzymes from *E. coli* and *A. thaliana* [11,12]. The same conserved residues are also found in SlDXS1 (His145, Asp245, Ile447, Glu449 and Phe474) and SlDXS2 (His141, Asp241, Ile443, Glu445 and Phe470) (Figure 4c). However, neither these conserved residues nor any predicted TPP binding site could be found in the SlDXS3 protein (Figure 4). Cl^-^ and pyruvate, rather than TPP, are the predicted ligands at the fourth binding site of SlDXS3 with residues Glu252, Arg549, Val251, Glu253, Glu420 and Lys250. Our results are in agreement with previous reports suggesting that the *A. thaliana* class 3 DXS (AtDXS3) cannot bind the TPP cofactor [17]. This might be the main reason explaining why SlDXS3 (and the other members of the class 3) lacks DXS activity.

### 2.4. SlDXS1 and SlDXS2 Co-Localize in Chloroplasts

**Figure 5:**
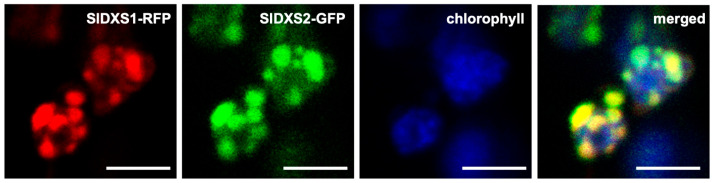
Subcellular localization of SlDXS1 and SlDXS2 fused to fluorescent proteins. Representative confocal microscopy images of chloroplasts from a *N. benthamiana* leaf cell transiently co-expressing SlDXS1-RFP and SlDXS2-GFP fusion proteins are shown. Panels show the same field under conditions to detect RFP fluorescence (red), GFP fluorescence (green), chlorophyll autofluorescence (blue) or all of them together (merged). Bar, 5 µm.

When fused to fluorescent proteins and overexpressed, DXS1 and DXS2 enzymes from different plants localize in the chloroplast stroma but also form speckles corresponding to aggregates, whereas class 3 DXS proteins were shown to form an intraplastidial filamentous-like network [17,36,37,38,39]. Such differential localization is consistent with the proposedly distinct functions of DXS3 proteins. We recently showed that, as expected for an active DXS, SlDXS1-GFP forms fluorescent speckles when transiently overexpressed in *Nicotiana benthamiana* leaves [40]. Here, we tested whether the other active DXS enzyme present in tomato, SlDXS2, also showed a similar subplastidial localization. Additionally, we asked whether the two enzymes co-localized in the same fluorescent spots when found together in the same chloroplast, a situation that occurs in tomato tissues expressing the two genes. The C-termini of full-length tomato SlDXS1 and SlDXS2 proteins were fused to RFP and GFP, respectively. The corresponding constructs (*35S:SlDXS1-RFP* and *35S:SlDXS2-GFP*) were then co-expressed via agroinfiltration in *N benthamiana* leaves (Figure 5). Agroinfiltrated leaves were used for direct observation under a confocal microscope to identify the fusion proteins based on their fluorescence. Both fusion proteins were localized in chloroplasts and showed a spotted distribution (Figure 5). Furthermore, the fluorescence signals from SlDXS1-RFP and SlDXS2-GFP fusion proteins were observed to overlap (Figure 5), demonstrating that they co-localize in these protein aggregates.

**Figure 6:**
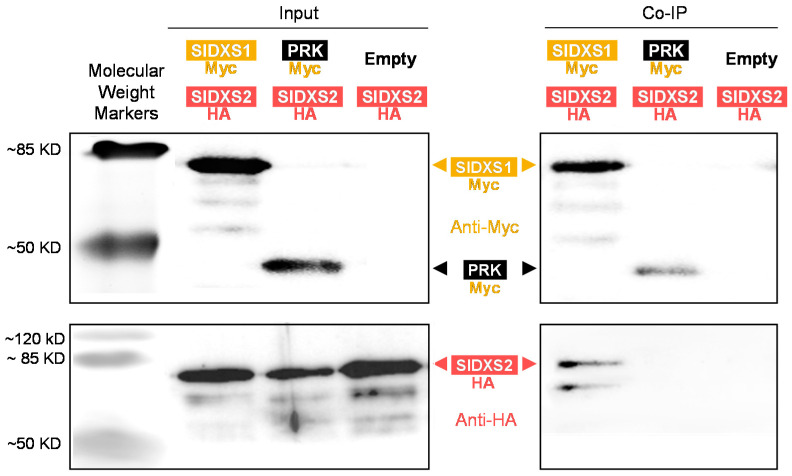
Co-immunoprecipitation assay. *N. benthamiana* leaves were co-agroinfiltrated with constructs to produce the indicated proteins (or an empty vector as a control). A fraction of the protein extracts was used to test protein production via immunoblot analyses with antibodies against Myc or HA (Input). After immunoprecipitation of the remaining protein extracts using anti-Myc, samples were used for immunoblotting analyses with anti-Myc (to confirm successful immunoprecipitation) and anti-HA (to detect the presence of co-immunoprecipitated HA-tagged proteins). The size of molecular weight markers (in KD) and the position of full-length epitope-tagged proteins are indicated. The rest of the bands likely correspond to incomplete/truncated/cleaved proteins or unspecific signals.

To confirm whether SlDXS1 and SlDXS2 could physically interact, co-immunoprecipitation assays were performed next (Figure 6). Instead of fluorescent proteins (known to dimerize themselves), for this experiment, we fused the C-terminal region of each tomato DXS isoform to smaller epitope tags. Constructs with SlDXS1 harboring a Myc tag (SlDXS1-Myc) and SlDXS2 with a hemagglutinin (HA) tag (SlDXS2-HA) were co-expressed in *N. benthamiana* leaves. As a control, we used a Myc-tagged version of *A. thaliana* phosphoribulokinase, a stromal enzyme of the Calvin cycle (PRK-Myc) [41]. After immunoprecipitation with an anti-Myc antibody, samples were analyzed voa immunoblot analysis using anti-Myc and anti-HA antibodies (Figure 6). The results conclusively showed that SlDXS2-HA could be co-immunoprecipitated using SlDXS1-Myc but not PRK-Myc, confirming that SlDXS1 and SlDXS2 can physically interact in vivo.

### 2.5. Heterodimers of SlDXS1 and SlDXS2 Can Be Potentially Active

**Figure 7:**
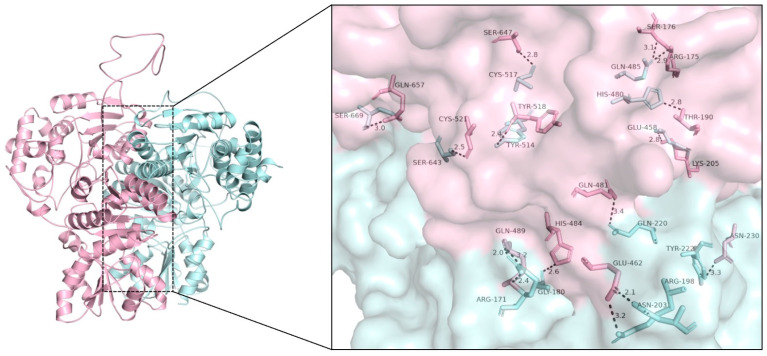
SlDXS1 and SlDXS2 heterodimer model. SlDXS1 monomer is shown in pink and SlDXS2 monomer in blue. Strong hydrogen bonds forming at the interface of the two monomers are shown in the magnification.

The co-localization and co-immunoprecipitation of SlDXS1 and SlDXS2 might be the consequence of the formation of enzymatically inactive co-aggregates of both proteins or, by contrast, reflect the existence of heterodimers. In order to investigate whether SlDXS1 and SlDXS2 might form enzymatically active heterodimers, the corresponding monomers were first computationally separated from their dimer structure by PyMOL and then used to create a virtual heterodimer with ZDOCK server [42]. The interaction structure model with the highest docking score (2619.573) was selected and then further analyzed with PyMOL. According to the ZDOCK result (Figure 7), the monomers of SlDXS1 and SlDXS2 can be arranged side by side to form a heterodimer that shows the same overall structure as SlDXS1 or SlDXS2 homodimers. The residues around the protein–protein interaction interface can form numerous hydrophobic interactions and hydrogen bonds that help stabilize the protein–protein complex (Figure 7).

## 3. Discussion

DXS catalyzes the main flux-controlling step of the MEP pathway and, hence, understanding how plants regulate this activity is key to design rationale-based biotechnological approaches aimed at increasing the production of plastidial isoprenoids in plant biofactories. The results reported in this paper show that from the three tomato genes encoding DXS-like proteins (SlDXS1, SlDXS2 and SlDXS3), only two appear to be true DXS enzymes (SlDXS1 and SlDXS2). As the housekeeping isoform contributing to the production of plastidial isoprenoids including carotenoids pigments [19,20], the *SlDXS1* gene is expressed at levels higher than *SlDXS2* in most tissues of the tomato plant (Figure 1). In particular, *SlDXS1* is strongly induced in the fruit pericarp during ripening, when MEP-derived precursors are needed to feed the carotenoid pathway for the production of lycopene and other pigments that eventually change the fruit color from green to red (Figure 1). The more restricted distribution of *SlDXS2* transcripts is in agreement with the more specialized roles proposed for class 2 DXS enzymes [13,18]. Interestingly, *SlDXS2* shows expression peaks during the development of flowers and fruits (Figure 1). In the case of fruits, *SlDXS2* expression peaks in the pericarp of young immature fruit (Figure 1b) and in maturing seeds (Figure 1c). Tomato seed development includes an initial phase of tissue differentiation followed by a second phase as the fruit expands involving the accumulation of nutrient reserves and acquisition of germination and desiccation tolerance [43]. Seeds achieve full germinability when fruits reach their final size at the MG stage, and then mature seeds dry concomitantly with fruit ripening. A transient accumulation of ABA supplied by the embryo during this last phase of seed maturation is key to acquire dormancy [43]. ABA is a carotenoid-derived hormone whose production in tomato seeds appears to be somehow controlled by specific isoforms of phytoene synthase (PSY), the enzyme catalyzing the first and main rate-determining step of the carotenoid pathway [44]. Of the two PSY-encoding isoforms expressed in tomato seeds, *SlPSY1* shows little to no expression in embryos and only *SlPSY2* was found to be required to produce ABA for seed dormancy [44]. It is possible that soon after the MG stage, SlDXS2 provides the precursors for eventually generating ABA via SlPSY2 in embryonic tissues. By contrast, SlDXS1 role in seeds might be more restricted to the seed coat at earlier stages of development (Figure 1). *SlDXS3* expression is higher than that of *SlDXS2* in embryo tissues, but the physiological relevance of this observation is unclear.

A coordinated role for SlDXS1 and SlDXS2 in the pericarp of young fruit could be deduced based on their overlapping expression pattern (Figure 1b) and their direct physical interaction (Figure 6), suggesting that they might be forming heterodimers. Based on the predicted structure of the homodimers (Figure 4) and the proposed heterodimers (Figure 7), it is very likely that the latter are enzymatically active. The observation that both SlDXS1 and SlDXS2 show DXS activity in *E. coli* (Figure 2) but SlDXS2 might be more prone to aggregation (Figure 3) supports the existence of isoform-specific features that are likely important for their regulation and, hence, for the control of the MEP pathway flux. Whether the catalytic activity or/and the stability of SlDXS1-SlDXS2 heterodimers is lower or higher than that of the corresponding homodimers remains to be investigated.

DXS3 proteins from *A. thaliana*, maize, melon and rice have been found to lack functional DXS activity in *E. coli* complementation assays [15,17]. Our data further provide both in vivo and in silico evidence that SlDXS3 is not a true DXS. The tomato SlDXS3 protein is unable to complement the growth of DXS-defective *E. coli* strains (Figure 2) or to enhance the MEP pathway flux in bacterial cells (Figure 3), in agreement with the observation that it lacks a binding site for the TPP cofactor that is essential for DXS activity (Figure 4). The possibility that SlDXS3 monomers could interact with SlDXS1 or SlDXS2 forming SlDXS1-SlDXS3 or SlDXS2-SlDXS3 heterodimers to decrease the DXS activity is difficult to reconcile with the pattern of expression of the *SlDXS3* gene (Figure 1). Indeed, it would be expected that *SlDXS3* expression would be more responsive to developmental (or environmental) cues modulating the request of IPP and DMAPP production. However, the virtually constitutive profile and low expression level of SlDXS3 argue against a major regulatory role for this protein by forming heterodimers with SlDXS1 and SlDXS2 isoforms to decrease their activity.

## 4. Materials and Methods

### 4.1. Bacterial Strains, Plant Material and Growth Conditions

*Escherichia coli* and *Agrobacterium tumefaciens* strains were grown in Luria broth (LB: 10 g/L bacto tryptone, 5 g/L yeast extract, and 10 g/L NaCl, plus 15 g/L bacto agar for plates) supplemented, when required, with relevant antibiotics as described in [40]. Bacterial growth in liquid media was monitored by measuring optical density at 600 nm (OD600). *Nicotiana benthamiana* plants were grown under standard greenhouse conditions.

### 4.2. Gene Constructs

The chloroplast-targeting motifs of SlDXS1, SlDXS2 and SlDXS3 proteins were predicted using Target P (https://services.healthtech.dtu.dk/service.php?TargetP-2.0 (accessed on 10 October 2018)). Then, the coding sequences lacking these N-terminal motifs were PCR-amplified from a ripe tomato fruit cDNA library using gene-specific primers (Table 2). The amplicons were ligated to pBluescript (pBSK) vectors in the presence of the restriction enzyme SmaI, which only cleaves the pBSK vectors lacking an insert. The generated constructs, pBSK-SlDXS1, pBSK-SlDXS2 and pBSK-SlDXS3, were used for expression in bacteria. To generate the constructs for expression in plants, the full-length coding sequences of SlDXS1 and SlDXS2 (with their chloroplast targeting peptides) were amplified from the cDNA library using specific primers (Table 2). Constructs pGWB454-SlDXS1-RFP (35S:SlDXS1-RFP), pGWB405-SlDXS2-GFP (35S:SlDXS2-GFP), pGWB420-SlDXS1-Myc (35S:SlDXS1-Myc) and pGWB414-SlDXS2-HA(35S:SlDXS2-HA) were obtained following a two-step (BP/LR) gateway reaction yielding proteins fused to C-terminal specific tags. All constructs were sequenced to confirm the identity of the genes and the absence of undesired mutations.

### 4.3. Complementation and Carotenoid Production Assays

Competent cells of the DXS-defective *E. coli* strain EcAB4-2 [22] were transformed with pBSK constructs and positive transformants were selected on solid LB medium supplemented with 1 mM MVA and appropriate antibiotics (kanamycin and ampicillin). To test for a functional DXS gene, transformed colonies were transferred to fresh media with or without MVA and incubated at 37 °C overnight. To analyze the effect of tomato DXS isoforms on *E. coli* carotenoid production, competent BL21(DE3) cells were co-transformed with plasmid p421-LYC [23] together with pBSK-SlDXS1, pBSK-SlDXS2, pBSK-SlDXS3 or an empty plasmid as a control. After the selection of transformants on LB plates supplemented with both chloramphenicol and ampicillin, several independent colonies were used to inoculate liquid cultures that were grown overnight at 37 °C and then diluted to a final density (OD600) of 0.05 in fresh LB plus chloramphenicol and ampicillin. These freshly inoculated cultures were grown at 37 °C until reaching an OD600 of 0.6. At this point, IPTG was added to a final concentration of 0.1 mM and the cultures were incubated at 30 °C for 24 h. Aliquots were then taken to measure OD600 and to estimate lycopene contents after extraction with acetone and quantification by measuring absorbance at 472 nm as described [45].

### 4.4. Computational Modeling

The full-length amino acid sequences of SlDXS1, SlDXS2 and SlDXS3 were uploaded to the Swiss-Model server (https://swissmodel.expasy.org/ (accessed on 9 April 2022)) and the protein structure with highest sequence identity was selected for the following analyses. To predict the protein binding site, Swiss-Model structures were uploaded to the 3D Ligand Site server (https://www.wass-michaelislab.org/3dlig/index.html (accessed on 25 April 2022)) and the binding sites were predicted by PyMOL. After separating dimeric SlDXS1 and SlDXS2 into monomers with PyMOL, SlDXS1 and SlDXS2 monomers were uploaded to the ZDOCK server (https://zdock.umassmed.edu/ (accessed on 25 April 2022)) and the complex with highest score was selected to analyze the hydrophobic interactions and hydrogen bonds.

### 4.5. Transient Expression Assays

*A. tumefaciens* GV3101 cells were transformed with the indicated constructs and transformants were selected on LB medium supplemented with rifampicin, gentamycin and spectinomycin. Single colonies were grown at 28 °C overnight in 3 mL of liquid medium and this preculture was used to inoculate 25 mL of fresh medium. After incubation at 28 °C overnight, the culture was centrifuged to collect cells and the pellet was resuspended in agroinfiltration buffer (10 mM MES pH = 5.6, 10 mM MgCl_2_ and 150 μM acetosyringone) to an OD600 of 0.8. The mixture was incubated at 28 °C for 1.5 h at 200 rpm and then mixed with 1/9 volumes of a culture carrying the helper component protease (Hc-Pro) of the Watermelon mosaic virus (WMV) in the vector pGWB702 to prevent gene silencing [41]. The mixture of the two cultures was infiltrated with a syringe in the abaxial part of leaves from 4- to 6-week-old *N. benthamiana* plants.

### 4.6. Subcellular Localization Assays

A 1:1 mix of 35S:SlDXS1-GFP and 35S:SlDXS2-RFP solutions was used for agroinfiltration. After 3 days, agroinfiltrated leaf tissue was observed with a Leica TCS SP8-MP Confocal Laser Scanning Microscope (Leica, Wetzlar, Germany). GFP fluorescence was detected using a BP515-525 filter after excitation at 488 nm, whereas the RFP signal was detected after excitation at 532 nm laser line and detected at 588 nm. A 610–700 nm filter was used to detect chlorophyll autofluorescence. All images were acquired using the same confocal parameters.

### 4.7. Co-Immunoprecipitation Assays

A 1:1 mix of *35S:SlDXS2-HA* and *35S:SlDXS1-Myc* or *35S:PRK-Myc* solutions was used for agroinfiltration. Leaf tissues were collected after 5 days, snap-frozen in liquid nitrogen, and stored at −80 °C until used for experiments. Protein extraction, immunoprecipitation, and immunoblot assays were carried out as described [41].

## Figures and Tables

**Table 1:** Results of protein modeling by Swiss-Model.

Gene	Template	Identity	Coverage	Range	Ramachandran Favoured	QMEAN	QMEANDisCo Global
*SIDXS1*	7bzx.1. A	88.75%	0.92	72–706	88.13%	0.72	0.73 ± 0.05
*SIDXS2*	7bzx.1. A	73.93%	0.91	68–702	87.28%	0.70	0.72 ± 0.05
*SIDXS3*	7bzx.1. A	58.24%	0.92	69–696	86.18%	0.65	0.68 ± 0.05

**Table 2:** Primer list for gene amplification.

Gene ID	Primer Name	Sequence (5′ to 3′)
*Solyc01g067890*	SlDXS1-F	ATCATGGCTTTGTGTGCTTATGCATTTCCTG
	SlDXS1-R	CTTACTCGAGTGTCATGACCTCTAGAGCCTCTC
*Solyc11g010850*	SlDXS2-F	CAGATGGCAGTTTCTTCAGGCACTGTATTTAG
	SlDXS2I-R	CTTAGTCGACGTTTACTACCATAGCTTCTTTTG
*Solyc08g066950*	SlDXS3-F	TGATGGTTGCTGTTACTGCTCAGTACCCATTTG
	SlDXS3L-R	GTTAGTCGACGCACATCAAAAGAAGAGCTTCTC
*Solyc01g067890*	GW-SIDXS1-F	GGGGACAAGTTTGTACAAAAAAGCAGGCTCGATGGCTTTGTGTGCTTATGCAT
	GW-SIDXS1-R	GGGGACCACTTTGTACAAGAAAGCTGGGTCTGTCATGACCTCTAGAGC
*Solyc11g010850*	GW-SIDXS2-F	GGGGACAAGTTTGTACAAAAAAGCAGGCTCGATGGCAGTTTCTTCAGGCACT
	GW-SIDXS2-R	GGGGACCACTTTGTACAAGAAAGCTGGGTCGTTTACTACCATAGCTTC

## Data Availability

Data are contained within the article.
